# The impact of upper motor neuron involvement on clinical features, disease progression and prognosis in amyotrophic lateral sclerosis

**DOI:** 10.3389/fneur.2023.1249429

**Published:** 2023-09-26

**Authors:** Eleonora Colombo, Francesco Gentile, Alessio Maranzano, Alberto Doretti, Federico Verde, Marco Olivero, Delia Gagliardi, Matteo Faré, Megi Meneri, Barbara Poletti, Luca Maderna, Stefania Corti, Massimo Corbo, Claudia Morelli, Vincenzo Silani, Nicola Ticozzi

**Affiliations:** ^1^Department of Neurology and Laboratory of Neuroscience, IRCCS Istituto Auxologico Italiano, Milan, Italy; ^2^Neurology Residency Program, Università degli Studi di Milano, Milan, Italy; ^3^“Dino Ferrari” Center, Department of Pathophysiology and Transplantation, Università degli Studi di Milano, Milan, Italy; ^4^Neurology Unit, Fondazione IRCCS Ca’ Granda Ospedale Maggiore Policlinico, Milan, Italy; ^5^Department of Neurology, San Gerardo Hospital ASST, Monza, Italy; ^6^School of Medicine and Surgery, Università degli Studi di Milano-Bicocca, Milan, Italy; ^7^Department of Neurorehabilitation Sciences, Casa di Cura Igea (CCI), Milan, Italy

**Keywords:** amyotrophic lateral sclerosis, motor neuron disease, Penn upper motor neuron score, upper motor neuron involvement, transcranial magnetic stimulation

## Abstract

**Objectives:**

In amyotrophic lateral sclerosis (ALS) both upper (UMNs) and lower motor neurons (LMNs) are involved in the process of neurodegeneration, accounting for the great disease heterogeneity. We evaluated the associations of the burden of UMN impairment, assessed through the Penn Upper Motor Neuron Score (PUMNS), with demographic and clinical features of ALS patients to define the independent role of UMN involvement in generating disease heterogeneity, predicting disease progression and prognosis.

**Methods:**

We collected the following clinical parameters on a cohort of 875 ALS patients: age and site of onset, survival, MRC scale, lower motor neuron score (LMNS), PUMNS, ALSFRS-R, change in ALSFRS-R over time (DFS), MITOS and King’s staging systems (KSS). Transcranial magnetic stimulation was performed on a subgroup of patients and central motor conduction time (CMCT) and cortical silent period (CSP) were calculated.

**Results:**

We observed that patients with an earlier age at onset and bulbar onset had higher PUMNS values. Higher values were also associated to lower ALSFRS-R and to higher DFS scores, as well as to higher MITOS and KSS, indicating that a greater UMN burden correlates with disease severity. Conversely, we did not appreciate any association between UMN involvement and survival or markers of LMN impairment. Moreover, PUMNS values showed a positive association with CMCT and a negative one with CSP values.

**Interpretation:**

Our results suggest that the burden of UMN pathology, assessed through PUMNS, has an important independent role in defining clinical characteristics, functional disability, disease progression and prognosis in ALS patients. We also support the role of TMS in defining severity of UMN involvement.

## Introduction

Amyotrophic lateral sclerosis (ALS) is a fatal neurodegenerative disease of adult life of unknown etiology, predominantly affecting the motor system, and caused by the progressive loss of motor neurons within the primary motor cortex (upper motor neurons, UMNs), the nuclei of the brainstem and the anterior horns of the spinal cord (lower motor neurons, LMNs). It is a heterogenous disorder with complex biology and significant variability in clinical presentation and prognosis. Addressing the disease heterogeneity is a challenging issue in clinical trials, in order to create personalized prognostic models and identify those patients for whom specific therapeutic strategies may be expected to be more successful (1, 2).

In this context, clinical phenotype, mainly determined by the extent and the spreading pattern of UMN and LMN involvement, has a great impact on the pattern and rate of ALS progression and overall survival prognosis (3).

However, few studies have analyzed the independent contribution of UMN vs. LMN signs on generating disease heterogeneity (3, 4), giving often great emphasis to LMN involvement alone (4, 5).

For these reasons, we focalised our analysis on the study of UMN signs, demonstrating that both clinical and instrumental evaluations can be useful in defining severity of UMN involvement.

Over time, different clinical scales (e.g., MGH UMNS or UK UMN) measuring hyperreflexia alone have been proposed to quantify UMN dysfunction (6). In our report we decided to use the Penn Upper Motor Neuron Score (PUMNS) that offers a more in-depth characterization of clinical UMN signs, as well as a more accurate correlation with functional disability, since it measures spasticity and pseudobulbar affect in addition to hyperreflexia and it is balanced to evaluate all segments of the body (7).

Here, we aim to evaluate the associations of the burden of UMN impairment, clinically assessed through PUMNS, with demographic and clinical features of ALS patients to define the role of UMN involvement in generating disease heterogeneity and predicting disease progression and prognosis.

Furthermore, we studied the correlations between PUMNS values and neurophysiological parameters of transcranial magnetic stimulation (TMS) to confirm whether PUMNS represents a reliable marker of UMN impairment and support the role of TMS in defining severity of UMN involvement.

## Patients and methods

### Patients

We recruited between 2008 and 2021 at IRCCS Istituto Auxologico Italiano a cohort of patients diagnosed with motor neuron disease [ALS, primary lateral sclerosis (PLS) and progressive muscular atrophy (PMA)] according to the El Escorial revised criteria (8). The following demographic and clinical data were collected: sex; age at onset; survival; site of onset; ALSFRS-R score at evaluation (first visit); progression rate, defined by the change in ALSFRS-R over time (deltaALSFRS-R, DFS) calculated with the formula [(48 – ALSFRS-R score)/disease duration expressed in months]; clinical stages according to the Milano-Torino (MITOS) and King’s staging systems; presence of mutations in *C9orf72*, *SOD1*, *TARDBP*, and *FUS* genes. Clinical phenotypes were classified according to Chio et al. into 8 groups (9): classic ALS; bulbar phenotype, characterized by isolated bulbar involvement for the first 6 months from onset; UMN-predominant (UMNp) ALS, dominated by spastic para−/tetraparesis together with other pyramidal signs over wasting and weakness, which are anyway detectable on clinical examination; PLS and PMA, characterized by pure UMN and LMN signs, respectively; flail arm phenotype, with progressive, predominantly proximal, LMN involvement of the upper limbs, remaining isolated in the cervical region for at least 12 months; flail leg phenotype, where wasting and weakness start on distal lower limbs and then spread progressively to other regions; and respiratory phenotype, in which dyspnoea and orthopnoea are the presenting symptoms (9). Patients with any another coexisting neurological conditions, such as other diseases affecting UMNs, were excluded from our analysis.

### Motor phenotyping

The burden of clinical UMN signs in ALS patients was assessed using the PUMNS. The PUMNS scale was designed using data obtained from approximately 1800 patients seen at a tertiary ALS center and modifying previous existing scales for the evaluation of UMN dysfunction (10). The instrument evaluates the presence and distribution of brisk deep tendon reflexes, as well as pathological reflexes routinely assessed during the neurological examination of ALS patients. It also includes information derived from two previously validated scales, namely the CNS-Lability Scale for measurement of pseudobulbar affect, and the Ashworth Spasticity Scale to assess limb spasticity (11–14). PUMNS has been proposed as a quick and easy- to-perform score, balanced to evaluate all segments of the body, with high accuracy and intra- and inter-rater reliability. The scale ranges from 0 to 32 (0–4 for the bulbar segment, 0–7 for each limb), with higher scores corresponding to greater UMN burden. For the bulbar region one point is given each for jaw jerk, facial reflex, palmomental reflex and a score ≥ 13 on the CNS-Lability Scale, a questionnaire evaluating pseudobulbar affect symptoms (11, 12). In the limbs one point is assigned each for a pathologically brisk reflex (triceps, biceps, finger flexor, patellar, crossed adduction, Achilles), and presence of Hoffmann and Babinski signs or clonus. A score ranging from 0 to 2 may be given for each limb based on the modified Ashworth Spasticity Scale (13, 14). The PUMNS was calculated for each patient from the clinical records.

The burden of LMN involvement was assessed using Lower Motor Neuron Score (LMNS), quantifying weakness and wasting in each limb on a scale from 0 to 3 (4). We modified the scale to consider the presence of LMN impairment also in the thoracic and bulbar regions, assigning 1 point each, for a maximum LMNS of 14. Weakness of spinal muscles was also evaluated using the MRC scale, assessing the strength of three muscle groups for each limb (shoulder abductors, elbow flexors, wrist dorsiflexors for the upper limbs; hip flexors, knee extensors and ankle dorsiflexors for the lower limbs) for a total score of 0–60.

The presence of ocular movement abnormalities was defined by the evidence at the neurological examination of at least one of the following alterations: saccadic dysfunction, smooth pursuit gain reduction; isolated upward gaze limitation; ocular apraxia; conjugate gaze palsy (15).

### Neurophysiological assessment

A subgroup of patients was also studied with transcranial magnetic stimulation (TMS). The analysis was performed with a Magstim Super Rapid^2^ Plus equipped with a 9 cm monophasic circular coil. The responses Motor Evoked Potentials (MEP) and the Cortical Silent Period (CSP) were recorded by electromyograph X1 – Nihon Kodhen. A compound muscle action potential (cMAP) was obtained by magnetic stimulation of the cervical/lumbar roots and measuring the F response. The coil of the magnetic stimulator was then placed on the contralateral hemisphere and moved slightly to determine the optimal position on the scalp where to obtain a motor evoked potential (MEP) of maximum amplitude (hot spot). The evoked responses were acquired with an acquisition time of 50 msec, an amplification ranging from 1 to 5 mV and with 5 kHz-10 Hz digital filters. MEP and CSP considered in the study were therefore obtained by delivering a magnetic stimulus equal to the threshold intensity +30%. The MEP was assessed by calculating the peak-to-peak amplitude and the average trace duration of two acquisitions obtained with relaxed muscle and three acquisitions during facilitation with mild voluntary muscle activation.

Central Motor Conduction Time (CMCT) was calculated by subtracting the average peripheral conduction time from that obtained after cortical stimulus. To obtain CSP for the upper limbs the following parameters were used: acquisition time of 200 msec, a variable amplification from 200 to 500 μV and with 5KHz-10 Hz digital filters. CSP was then calculated according to already described protocols (16, 17).

Individual PUMNS subscores obtained from each limb (right arm, left arm, right leg, left leg) were compared to CMCT and CSP derived from the corresponding region. Total PUMNS was also compared to CMCT and CSP values.

### Genetic screening

Genomic DNA was extracted from peripheral blood using the Wizard Genomic DNA Purification Kit (Promega). The entire coding region of *SOD1*, exon 6 of *TARDBP*, and exons 5, 6, 13, 14 and 15 of *FUS*, as well as the intron/exon boundaries were amplified by PCR using custom-made primers and directly sequenced using BigDyeTerminator v3.1 cycle sequencing kit on an ABI PRISM 3700 Genetic Analyzer (Applied Biosystems). Genetic analysis of *c9orf72* was performed using a two-step PCR protocol, as previously described (18). For hexanucleotide repeat expansion determination, fluorescent fragment length analysis with flanking primers was performed on an ABI PRISM 3700 Genetic Analyzer and data were visualized using GeneMapper v4.0 software (Applied Biosystems).

Samples showing a single peak were further tested for the presence of hexanucleotide repeat expansions by repeat-primed PCR. A cutoff value of >30 repeats was used to define the pathogenic threshold.

### Statistical analysis

Descriptive statistics are reported as numbers and percentages for categorical variables or mean, median and standard deviation for continuous variables. Survival analysis was performed dividing patients in two groups according to the median value of PUMNS and building Kaplan–Meier curves with their median survival and 95% confidence interval (95% CI). The log-rank test was used to compare survival across groups. We compared PUMNS values with categorical variables using Kruskal-Wallis K test and with continuous variables using Pearson correlation. A *p* < 0.05 was considered significant, and all tests were two-sided. Statistical analysis was carried out with IBM Statistical Package for Social Science (SPSS) version 26.

### Standard protocol approval and patient consent

Informed consent for using anonymized data for research purposes was obtained from all patients or their authorized legal representatives. Anonymized data are archived on Zenodo 10.5281/zenodo.6617178 and will be disclosed upon reasonable request. This study was approved by the Ethics Committee of IRCCS Istituto Auxologico Italiano (2021_05_18) and conducted according to the principles expressed in the Declaration of Helsinki.

## Results

### Cohort description

We recruited a cohort of 871 patients, 329 (37.8%) of whom were females and 542 (62.2%) males. The mean age of disease onset and median survival were, respectively, 59.8 years (± 12.3) and 51.7 months (95% CI 45.6–57.8). The mean age at evaluation of patients was 61.9 years (± 11.8). Data, including PUMNS and neurophysiological assessment, were collected at the same time, during the first visit in our center. The median time to visit was 14.7 months after disease onset (range 1.5–273.7). In particular, the median time to visit after disease onset, expressed in months, was 12.0 (range 1.7–196.2) for bulbar phenotype, 13.7 (range 1.5–175.7) for classic ALS, 23.5 (range 3.0–273.7) for flail arm patients, 27.0 (range 4.2–207.6) for flail leg phenotype, 40.1 (range 2.6–178.8) for PLS patients, 29.2 (range 3.9–227.9) for PMA individuals, 11.6 (range 3.5–54.8) for respiratory phenotype and 12.2 (range 3.2-171.l) for UMNp patients. Site of onset was known for 871 patients and was bulbar in 202 (23.2%) and spinal in 669 (76.8%) of patients. With regard to the motor phenotype, we could divide the cohort into the following phenotypes: classic (*N* = 460, 52.6%), respiratory (*N* = 17, 1.9%), bulbar (*N* = 177, 20.2%) and UMN-predominant (*N* = 79, 9.0%) ALS, flail arm (*N* = 37, 4.2%) and flail leg (*N* = 20, 2.3%) syndromes, PLS (*N* = 40, 4.6%), and PMA (*N* = 41, 4.7%) (Table 1). In our cohort, SOD1 was mutated in 18 patients (2.1%), TARDBP in 16 individuals (1.8%), FUS in 5 patients (0.6%) and C9orf72 in 46 patients (5.3%). An ocular movement disorder is present in 78 (8.9%) individuals.

**Table 1:** Demographic and clinical characteristics of the study cohort.

	Total (*n* = 871)
Sex, female	329 (38%)
Age of onset, mean (SD)	59.8 (12.3)
Phenotypes	
Classic ALS	460 (53%)
Bulbar ALS	177 (20%)
UMNp ALS	79 (9%)
Flail arm	37 (4%)
Flail leg	20 (2%)
PLS	40 (5%)
PMA	41 (5%)
Respiratory	17 (2%)
*C9orf72* expansion (*n* = 822)	46 (5.6%)
Time to in-hospital admission, median (*r*)	14.7 (1.5–274)
ALSFRS-R, median (*r*)	39 (4–48)
ΔFS, median (*r*)	0.60 (0.05–7.9)
Survival, months	51.7 (45.6–57.9)
PUMNS, median (*r*)	9 (0–29)
LMNS, median (*r*)	4 (0–14)

Data are presented as *n* (%), mean (SD) or median (range) where appropriate. LMNS, lower motor neuron score; PLS, primary lateral sclerosis; PMA, progressive muscular atrophy; PUMNS, Penn’s UMN score; UMNp, UMN-predominant ALS; UMN, upper motor neuron.

### Association of UMN involvement with clinical phenotype, disability and survival

**Figure 1:**
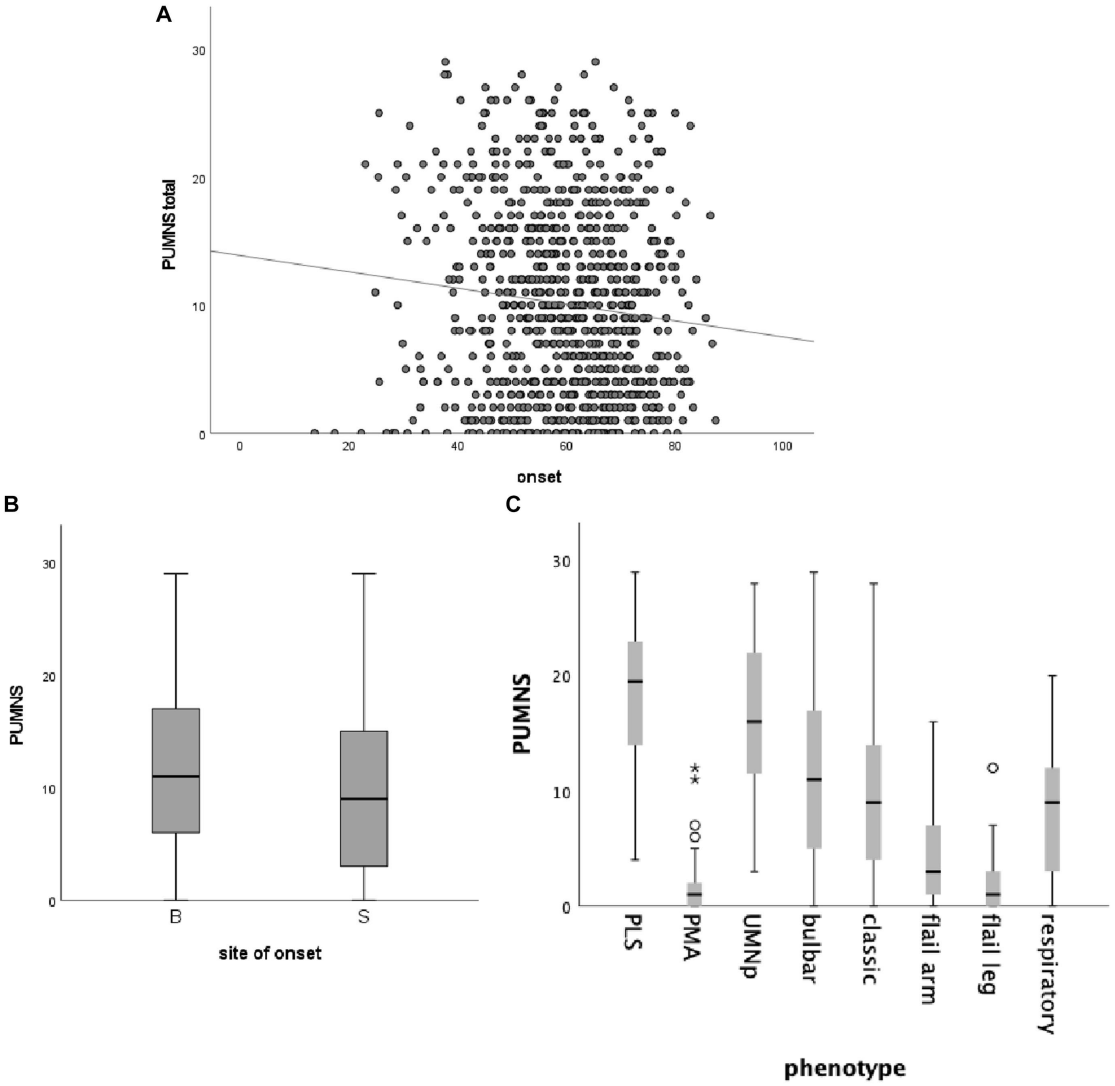
Correlation between total PUMNS values, age at onset **(A)**, site of onset **(B)** and motor phenotype **(C)**. **(A)** Scatter plot showing the correlation between total PUMNS values and age at onset (*R*^2^ = 0.011; *p* = 0.002). Each gray circle represents an ALS patient. Trend line is shown in black. **(B)** Box plot showing the correlation between total PUMNS values and site of onset (*p* = 2.3×10^−4^). For each group the bold line shows the median, the gray boxes include the middle 50% of the data and whiskers show the minimum and maximum values. B, bulbar; S, spinal. **(C)** Box plot showing the correlation between total PUMNS values and motor phenotype. For each group the bold line shows the median, the gray boxes include the middle 50% of the data and whiskers show the minimum and maximum values. Empty circles represent outliers (below Q1−1.5IQR and above Q3+1.5IQR), asterisks represent extreme outliers (below Q1−31QR and above Q3+3IQR).

In our cohort, we found an association between age at onset and PUMNS, with higher scores in patients with an earlier onset (*r* = −0.11; *p* = 0.002) (Figure 1A). Additionally, patients with bulbar onset had significantly higher PUMNS values compared with spinal-onset individuals (11 [0–29] vs. 9 [0–29]; *p* = 2.3×10^−4^) (Figure 1B). Similarly, patients with bulbar signs (*n* = 660) had significantly higher total (11 [0–29] vs. 4 [0–23]; *p* < 0.001) and spinal PUMNS (9 [0–26] vs. 4 [0–23]; *p* = 1.8×10^−12^) compared to individuals with only spinal signs (*n* = 211). Comparing patients by site of onset, bulbar-onset cases were strictly clustered in those phenotypes characterized, by definition, by a high UMN burden (classic ALS, bulbar palsy, UMNp ALS and PLS) while none of them belonged to those groups with predominant/pure LMN signs (Table 1). We observed significant differences in the distribution of PUMNS values among different motor phenotypes (Figure 1C). In particular, compared to classic ALS phenotype, PLS and UMNp patients displayed higher PUMNS values (20 [4–29] and 16 [3–28] vs. 9 [0–28]; *p* = 5.9×10^−9^ and *p* = 9.0×10^−11^, respectively), while individuals with PMA, flail arm or flail leg phenotypes had lower values compared to classic ALS (1 [0–12], 3 [0–16] and 1 [0–12] vs. 9 [0–28]; *p* = 2.6×10^−12^, *p* = 8.8×10^−5^ and *p* = 7.0×10^−6^, respectively). No differences were observed between classic and bulbar ALS phenotypes, as well as between PMA, flail arm and flail leg variants. Interestingly, higher PUMNS values appeared to correlate with the presence of eye movement dysfunction (12.3 ± 7.6 vs. 9.7 ± 7.2; *p* = 0.005), confirming our previous observation that these abnormalities are associated with an increased burden of UMN signs (15).

We then evaluated whether PUMNS is associated to ALSFRS-R and DFS values in order to assess the role of UMN involvement on functional disability and disease progression, respectively. We observed a significant association of higher PUMNS values with lower ALSFRS-R scores (*r* = −0.27; *p* = 3.5×10^−11^), as well as with higher DFS (*r* = 0.16; *p* = 7.3×10^−5^) in the whole cohort, indicating that a greater UMN burden correlates with disease severity. The LMN score also showed a significant negative correlation with ALSFRS-R (*r* = −0.68, *p* = 2.15×10^−81^) and a positive correlation with DFS (*r* = 0.21, *p* = 6.58×10^−7^). On linear regression analysis, we observed that both PUMNS (*β* = −0.25 ± 0.03, *p* = 8.9×10^−15^) and LMNS (*β* = −1.65 ± 0.07, *p* = 8.25×10^−85^) are independent predictors of functional disability. Interestingly, we did not find any association of PUMNS with disease duration at evaluation (*r* = −0.04, *p* = 0.2), nor with MRC (*r* = 0.02, *p* = 0.6) or LMNS values (*r* = 0.04, *p* = 0.26), suggesting that the progression of UMN and LMN impairment occurs at least partially independently during the course of the disease and that the increasing burden of LMN pathology does not significantly mask the presence of UMN signs.

**Figure 2:**
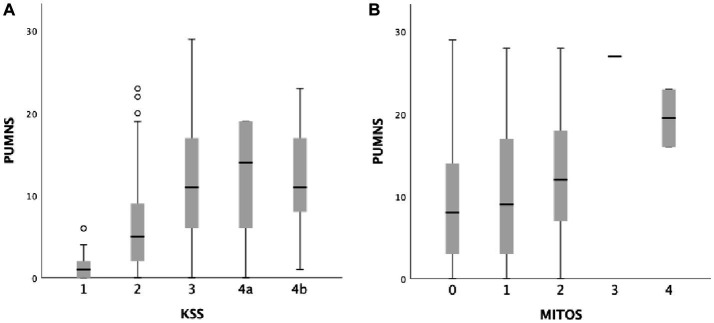
Box plots showing the correlation between total PUMNS values with KSS **(A)** and MITOS stages **(B)**. For each group the bold line shows the median, the gray boxes include the middle 50% of the data and whiskers show the minimum and maximum values. Empty circles represent outliers (below Q1−1.5IQR and above Q3+1.5IQR).

With regard to disease staging, in our cohort, higher PUMNS values were associated with increasing MITOS and King’s stages (Figure 2). In particular, for KSS we observed statistically significant differences when comparing stages 1 to 2 (1 [0–6] vs. 5 [0–23]; *p* = 2×10^−6^), 2 to 3 (5 [0–23] vs. 11 [0–29]; *p <* 10^−85^) and 2 to 4 (5 [0–23] vs. 12 [0–23]; *p* = 4.7×10^−5^). Conversely, for MITOS we could identify differences only when comparing patients at stages 0 and 2 (8 [0–29] vs. 12 [0–28]; *p* = 0.019).

**Figure 3:**
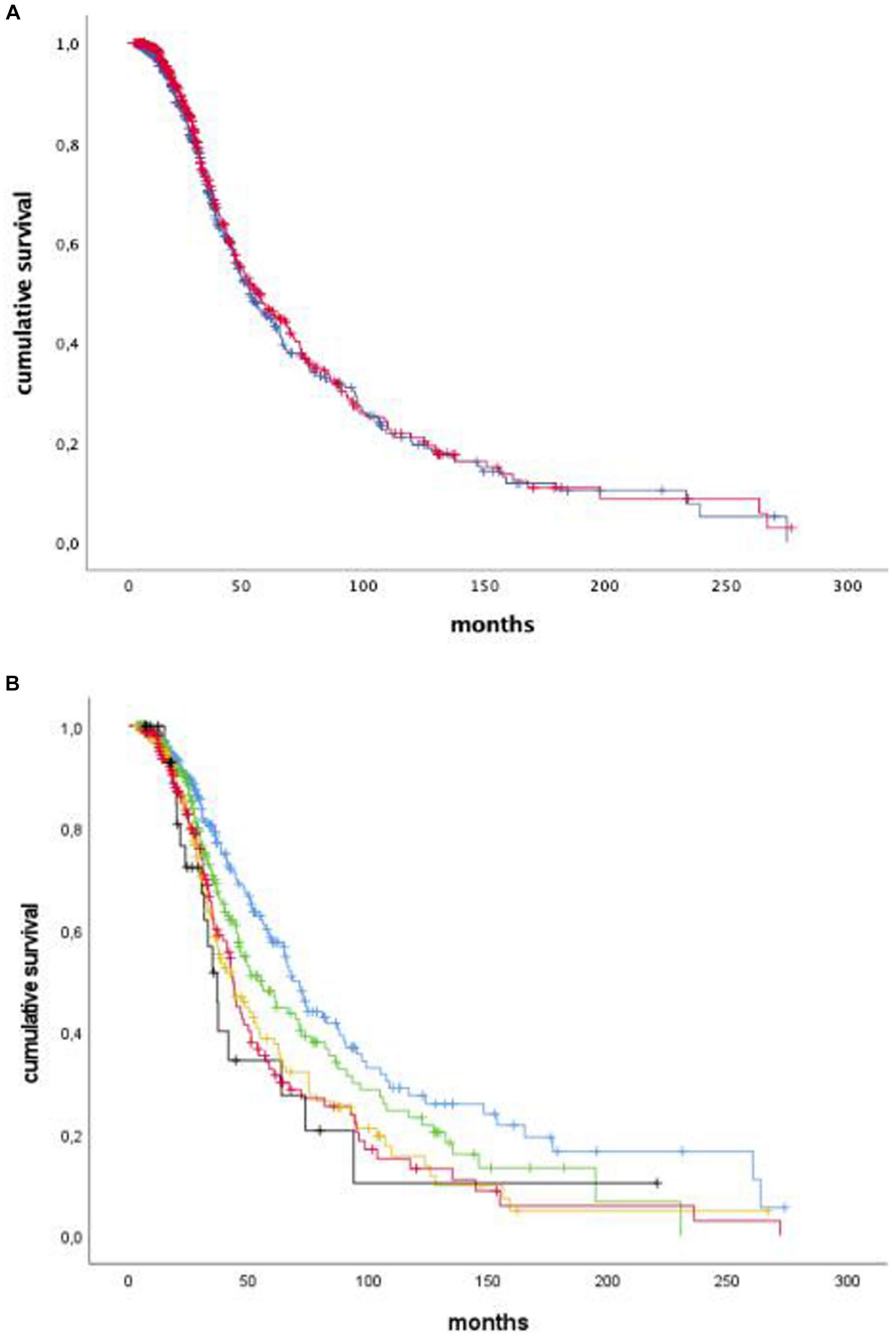
Kaplan–Meier curves of survival probabilities for ALS patients with total PUMNS values above (blue) or below (red) the median value of 9.0. We did not appreciate any association with survival (p-not significant: **A**). Kaplan–Meier curves of survival probabilities for ALS patients with bulbar PUMNS values of 0 (blue), 1 (green), 2 (yellow), 3 (red) and 4 (black). We found that patients without bulbar UMN impairment had a significantly prolonged survival (68.4 months) compared to patients with a score of 1 (55.4 months; p-0.047), 2 (43.5 months; *p* = 1.5 × 10^−4^), 3 (43.3 months; *p*=8.3 × 10^−5^) or 4 (36.7 months, *p* = 0.006: **B**).

After subdividing our cohort into two groups with PUMNS values above and below the median (9.0), respectively, we did not appreciate any association with survival (Figure 3A). Similar results were observed when we conducted separate survival analyzes for individual clinical phenotypes, according to the intra-group median PUMNS. Conversely, when considering the bulbar PUMNS subscore alone, we found that patients without bulbar UMN impairment had a significantly prolonged survival (68.4 months) compared to patients with a score of 1 (55.4 months; *p* = 0.047), 2 (43.5 months; *p* = 1.5×10^−4^), 3 (43.3 months; *p* = 8.3×10^−5^) or 4 (36.7 months; *p* = 0.006) (Figure 3B). Furthermore, we found that patients with at least a score of one at bulbar PUMNS subscore, and no LMN impairment, had a significant lower survival than patients without bulbar signs of disease (91 vs. 120 months, *p* = 0.014). This correlation increased in significance when we compared patients with both LMN and UMN signs in the bulbar segment to patients without bulbar involvement (77.4 vs. 120 months, *p* = 3.5×10^−5^).

Lastly, we investigated the degree of UMN involvement in patients carrying mutations in the four major ALS-associated genes, namely *c9orf72*, *SOD1*, *TARDBP* and *FUS*. Interestingly, we observed that patients carrying the *c9orf72* hexanucleotide repeat expansion had higher PUMNS values compared to individuals without the mutation (13 [0–28] vs. 9 [0–29]; p = 0.01), suggesting a phenotype characterized by UMN signs in this group. Conversely, *SOD1*-mutated cases had lower PUMNS values compared to the remaining ALS population (3 [0–14] vs. 9 [0–29]; *p* = 4.0×10^−5^), consistent with previous literature describing ALS1 as a predominantly LMN disease (19–21). We did not appreciate any association between PUMNS and the presence of *TARDBP* (8 [1–14] vs. 10 [0–29]; *p* = 0.07) and *FUS* (6 [4–12] vs. 11 [0–29]; *p* = 0.21) mutations.

### Association of UMN involvement with neurophysiological markers

**Figure 4:**
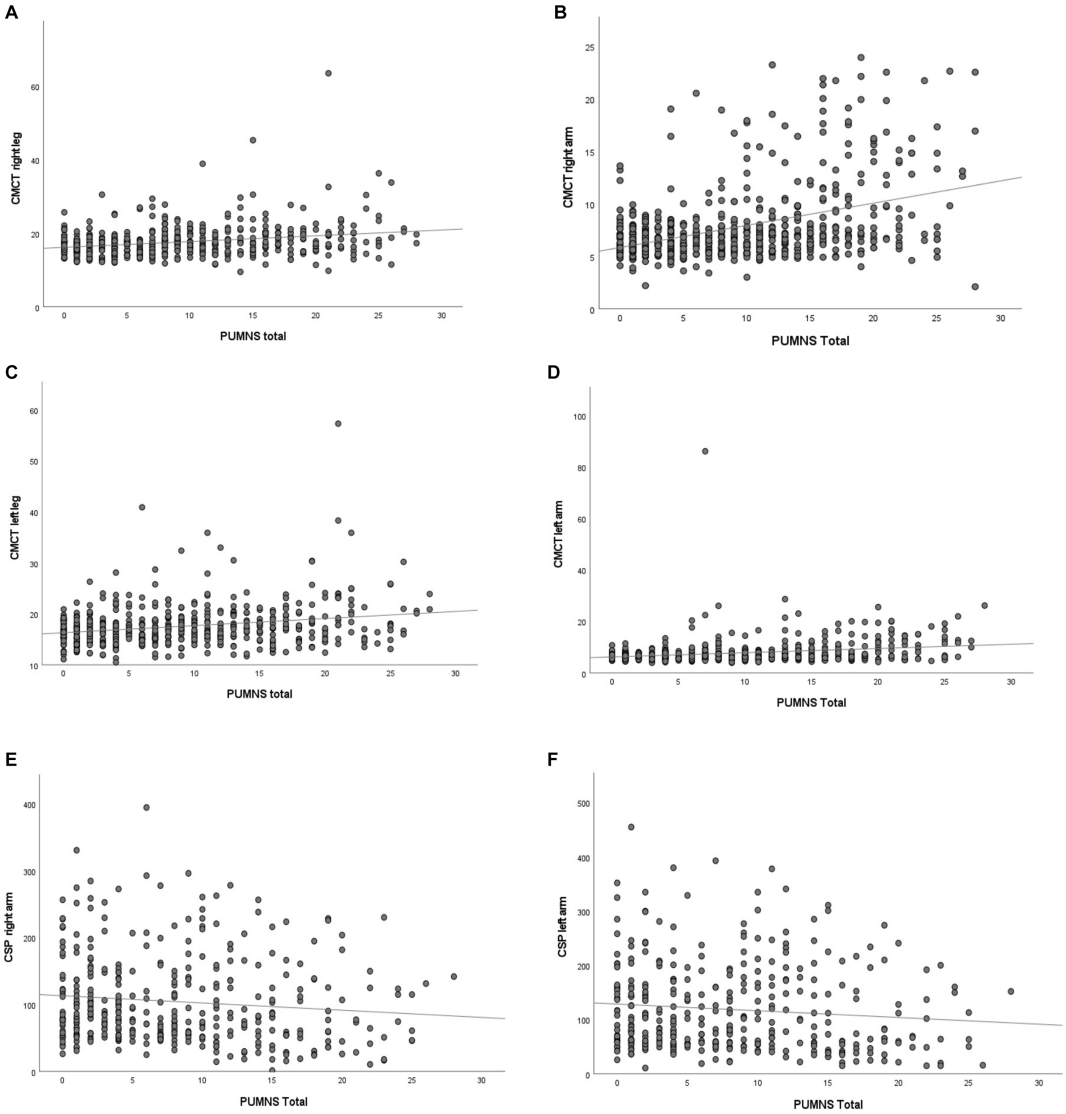
Scatter plot showing the correlation of total PUMNS values with CMCT values derived for the right arm [*R*^2^ = 0.167, *p* = 3.5×10^−16^; **(A)**], left arm [*R*^2^ = 0.121, *p* = 2.8×10^−18^; **(B)**], right leg [*R*^2^ = 0.066, *p* = 2.0×10^−8^; **(C)**] and left leg [*R*^2^ = 0.059; *p* = 2.0×10^−6^; **(D)**], as well as with CSP values derived from the right [*R*^2^ = 0.012, *p* = 0.011; **(E)**] and left arm [*R*^2^ = 0.010, *p* = 0.024; **(F)**]. Each gray circle represents an ALS patient. Trend line is shown in black. CMCT, central motor conduction time; CSP, cortical silent period.

We performed TMS and obtained the CMCT for the four limbs on a subset of 615 (70.3%) patients of our cohort. We observed an association between partial PUMNS scores calculated for each limb and CMCT values derived from the corresponding region (right arm: *r* = 0.42, *p* = 7.8×10^−28^; left arm: *r* = 0.28, *p* = 2.6×10^−12^; right leg: *r* = 0.30, *p* = 7.3×10^−13^; left leg: *r* = 0.28, *p* = 4.8×10^−11^). Total PUMNS was also associated with higher CMCT values in all limbs (right arm: *r* = 0.41, *p* = 3.24×10^−26^; left arm: *r* = 0.25, *p* = 1.6×10^−9^; right leg: *r* = 0.26, *p* = 1.1×10^−9^; left leg: *r* = 0.24; *p* = 8.66×10^−9^) (Figures 4A–D).

Within the group of ALS patients studied with TMS, we could derive CSP values for the upper limbs in 367 individuals. In this subset of patients, we observed that higher partial PUMNS for the right and left arm were associated with significantly lower CSP values derived from the corresponding limb (right arm: *r* = −0.16, *p* = 0.003; left arm: *r* = −0.12, *p* = 0.026). Finally, total PUMNS negatively correlated with right but not left CSP values (right arm: *r* = −0.11, *p* = 0.036; left arm: *r* = −0.10, *p* = 0.054) (Figures 4E,F). These data suggest that there might be a correlation between the clinical burden of UMN signs measured with PUMNS and neurophysiological markers of UMN dysfunction.

## Discussion

We investigated the correlations between the burden of UMN signs, measured through PUMNS, and clinical and neurophysiological features to analyze the independent contribution of UMN involvement in defining phenotype, disability, predicting disease progression and prognosis in ALS patients.

With regard to the clinical presentation, as expected, we observed higher PUMNS values in patients with phenotypes characterized by prominent UMN involvement (PLS and UMNp), compared to classic and bulbar ALS and to predominantly LMN diseases (flail arm and flail leg syndromes and PMA). These results suggest that this scale is a valuable marker of UMN impairment even if by itself does not directly assess the degree of functional disability due to UMN impairment.

Higher PUMNS values were also associated in our cohort with an earlier age at onset, confirming previous literature evidence that younger ALS patients have a phenotype characterized by more UMN impairment (9, 22, 23).

Moreover, we observed that patients with bulbar onset of disease had higher PUMNS.

We also found higher PUMNS in ALS patients presenting with different types of eye movement abnormalities, confirming our previous observation that oculomotor dysfunction occurs more frequently in phenotypes characterized by prominent UMN signs, compared to classic ALS and LMN diseases (15). Lastly, genetic subtypes of ALS appear to be associated with distinct phenotypes characterized by higher (c*9orf72*) or lower (*SOD1*) PUMNS values. In fact, it is well established that patients carrying *SOD1* mutations show clinical phenotypes characterized by predominant LMN impairment (18–20). Conversely, we did not find any association between the presence of *TARDBP* mutations and PUMNS, confirming the observation that these patients have phenotypes usually indistinguishable from classic ALS (24, 25). Lastly, while *FUS* mutations have been mainly associated to rapidly progressive, early-onset LMN diseases (26–28), we could not detect any difference in our cohort, presumably due to the small number of mutated patients.

It is of particular interest that we did not find in our cohort any association between the burden of UMN involvement and markers of LMN dysfunction (i.e., MRC and LMNS), suggesting that UMN and LMN pathologies progress at least partly independently from each other during the disease course in ALS patients. This finding could also indicate that the increasing burden of LMN signs does not necessarily mask the UMN disease to the point of significantly impairing the capability of PUMNS to measure UMN signs. Moreover, this scale appears to be a reliable tool not only at initial stages, but also in advanced disease stages. In fact, we observed a good correlation between higher PUMNS and more advanced KSS and MITOS stages, indicating that there is not a progressive loss of validity and informativeness during disease course.

Quantitative indicators of disease severity and progression are essential outcome measures in ALS patients, and they are often used to assess effects of interventions in randomized clinical trials. In this context, the ALSFRS-R is the most widely validated and broadly used instrument (29, 30), but very few studies have analyzed the independent contribution of UMN vs. LMN signs to functional disability and disease evolution (3–5, 31).

Similarly to a previous study (7), we found that PUMNS values negatively correlated with ALSFRS-R scores, indicating that UMN dysfunction significantly contributes to functional disability in patients with motor neuron diseases. Furthermore, we observed a direct correlation between PUMNS and DFS, suggesting that the burden of UMN signs can also be a good marker of disease progression rate.

The findings of our analysis also indicate that UMN impairment contributes to functional disability and disease progression irrespective of the presence of LMN signs.

Conversely, we did not appreciate any association between PUMNS and survival, suggesting that the global burden of UMN signs does not have a major role in determining disease prognosis, which is likely dependent on the severity of LMN pathology alone (4, 5). Our analysis indicates that UMNs are mainly involved in fine motor regulation and their impairment could lead to functional disability without causing severe weakness, which instead is secondary to LMNs degeneration.

It should be noted that analyzing the PUMNS bulbar subscore alone, we observed a strong correlation with survival, suggesting that UMN signs in the bulbar region, even in patients without any bulbar LMN involvement, indeed represent a negative prognostic factor.

Comparison with neurophysiological and neuroradiological parameters could be useful to confirm the efficacy of PUMNS in identifying UMN involvement. A prior study has demonstrated that higher PUMNS values were associated with diffusion tensor imaging metrics of disease progression and more extensive corticospinal tract pathology (32), suggesting that the scale is a reliable marker of UMN impairment. In our report we analyzed the correlation between PUMNS and TMS-derived parameters of cortical motor neuron and long-tract degeneration (CMCT), as well as cortical hyperexcitability and impaired inhibition (CSP). We found that PUMNS subscores obtained from each limb were positively associated to CMCT and negatively associated to CSP values derived from the corresponding region. Similar results were obtained for total PUMNS. Our data, together with previous clinical and radiological studies (7, 32), suggest that PUMNS is a reliable proxy of UMN pathology and correlates well with other such biomarkers.

Simpler clinical scales (e.g., MGH UMNS) measuring hyperreflexia alone have been proposed to quantify UMN dysfunction (6). Although such tools correlate well with UMN molecular imaging changes in ALS (33), PUMNS offers a more in-depth characterization of clinical UMN signs, as well as a more accurate correlation with functional disability, since it measures spasticity and pseudobulbar affect in addition to hyperreflexia. However, in the absence of further validation studies, this scale cannot be used as an independent tool for assessing prognosis or therapeutic response in randomized clinical trials.

Our study has some limitations. Firstly, TMS could be performed only on 70% of the study population. Several factors, such as poor patient collaboration or medication with skeletal muscle relaxants, may theoretically hinder the detection of UMN signs. Additionally, the evaluation of ALS patients was performed by several clinicians over the years and no inter-rater reliability analysis could be performed. Ours is a cross-sectional study and our cohort includes a relatively small number of patients at more advanced disease stages. For this reason, it is not possible to evaluate the longitudinal dynamics of PUMMS and to determine with certainty to what degree LMN signs mask UMN involvement as the disease progresses. Therefore, longitudinal data on independent cohorts will be needed to address this point.

Lastly, even if the overall ALS cohort is large, some phenotypes are rare, with only a limited number of affected patients, somewhat limiting the validity of PUMNS in these subgroups compared to the prevailing classical and bulbar ALS phenotypes.

Notwithstanding these limitations, it should be noted that in our cohort PUMNS appears to correlate with advanced disease stages as measured with KSS and MITOS and does not seem to be influenced by the increasing burden of LMN signs.

Our results suggest that the burden of UMN pathology has an important independent role in defining clinical characteristics, functional disability, disease progression and prognosis in patients affected by ALS. Moreover, this is the first study attempting to correlate PUMNS with neurophysiological parameters of UMN pathology in a large ALS cohort, although longitudinal data on independent ALS populations will be needed to confirm our results.

## Data availability statement

The datasets presented in this study can be found in online repositories. The names of the repository/repositories and accession number(s) can be found at: 10.5281/zenodo.6617178.

## Ethics statement

The studies involving humans were approved by IRCCS Istituto Auxologico Italiano. The studies were conducted in accordance with the local legislation and institutional requirements. The participants provided their written informed consent to participate in this study.

## Author contributions

EC contributed to the study design, data collection and interpretation, and first draft manuscript. FG, AD, AM, SC, and FV contributed to data collection and interpretation and review of the intellectual contents. MO, DG, MF, MM, LM, MC, CM, BP contributed to data collection. VS contributed to review of the intellectual contents. NT originally conceived study design, contributed to data interpretation, review of the intellectual contents and approved final manuscript. All authors contributed to the article and approved the submitted version.

## Funding

This work was financially supported by the Italian Ministry of Health – Ricerca Corrente to IRCCS Istituto Auxologico Italiano.

## Conflict of interest

VS received compensation for consulting services and/or speaking activities from AveXis, Cytokinetics, Italfarmaco, Liquidweb S.r.l., Novartis Pharma AG and Zambon. Receives or has received research supports form the Italian Ministry of Health, AriSLA, and E-Rare Joint Transnational Call. He is in the Editorial Board of Amyotrophic Lateral Sclerosis and Frontotemporal Degeneration, European Neurology, American Journal of Neurodegenerative Diseases, Frontiers in Neurology, and Exploration of Neuroprotective Therapy. NT received compensation for consulting services and/or speaking activities from Amylyx Pharmaceuticals, Italfarmaco and Zambon Biotech SA. He received research funding from the Italian Ministry of Health and AriSLA. He is Associate Editor for Frontiers in Aging Neuroscience.

The remaining authors declare that the research was conducted in the absence of any commercial or financial relationships that could be construed as a potential conflict of interest.

## Publisher’s note

All claims expressed in this article are solely those of the authors and do not necessarily represent those of their affiliated organizations, or those of the publisher, the editors and the reviewers. Any product that may be evaluated in this article, or claim that may be made by its manufacturer, is not guaranteed or endorsed by the publisher.
